# Rich do not rise early: spatio-temporal patterns in the mobility networks of different socio-economic classes

**DOI:** 10.1098/rsos.150654

**Published:** 2016-10-12

**Authors:** Laura Lotero, Rafael G. Hurtado, Luis Mario Floría, Jesús Gómez-Gardeñes

**Affiliations:** 1 Facultad de Ingeniería Industrial, Universidad Pontificia Bolivariana, Medellín, Colombia; 2 Departamento de Ciencias de la Computación y de la Decisión, Universidad Nacional de Colombia, Medellín, Colombia; 3 Departamento de Física, Universidad Nacional de Colombia, Bogotá, Colombia; 4 Departamento de Física de la Materia Condensada, Universidad de Zaragoza, Zaragoza 50009, Spain; 5 Instituto de Biocomputación y Física de Sistemas Complejos, Universidad de Zaragoza, Zaragoza 50018, Spain

**Keywords:** urban mobility networks, spatio-temporal patterns, socio-economic status

## Abstract

We analyse the urban mobility in the cities of Medellín and Manizales (Colombia). Each city is represented by six mobility networks, each one encoding the origin-destination trips performed by a subset of the population corresponding to a particular socio-economic status. The nodes of each network are the different urban locations whereas links account for the existence of a trip between two different areas of the city. We study the main structural properties of these mobility networks by focusing on their spatio-temporal patterns. Our goal is to relate these patterns with the partition into six socio-economic compartments of these two societies. Our results show that spatial and temporal patterns vary across these socio-economic groups. In particular, the two datasets show that as wealth increases the early-morning activity is delayed, the midday peak becomes smoother and the spatial distribution of trips becomes more localized.

## 1. Introduction

Understanding and modelling urban mobility is crucial for urban planning and decision-making and has been a topic of great interest for sociologists, urban planners, engineers, physicists, epidemiologists and others from decades ago [1–5]. The relationship between urban mobility, the transportation system and the development of cities is complex and multidimensional. It has been largely studied and there are well-establish models to represent urban mobility and its relationship with land use planning. However, in urban mobility, the underlying transportation system together with the individuals making use of it yield a complex system composed of multiple connected elements. The interaction between these elements produces nonlinear emergent behaviours. One of these collective phenomena is the emergence of congestion [6] owing to the interaction of a large number of individuals belonging to different areas of a city with the need to move to other parts of the city using common transportation means and pathways.

The recent availability of large datasets on human mobility, together with the explosion of computational power have allowed for new systematic studies of mobility patterns (in cities and worldwide), which have revealed important underlying general principles of mobility networks [7]. One plausible tool of complexity science to represent human mobility is the complex network formalism, which consists of mapping the elements of the system into nodes and links, forming networks. From its birth at the end of the last century [8], network science has evolved as a multidisciplinary benchmark garnering the interest of researchers from diverse scientific realms. The power of this approach relies on the efficient way it allows the encoding of information about the very many interactions between parts or elements to model and explain the mechanism for the emergence of collective behaviour [9–12].

In this work, we make use of the network formalism, in particular that of mobility networks, to analyse the spatio-temporal patterns of the movement of individuals in two cities of Colombia, Medellín and Manizales. We will take advantage of the high spatial and temporal resolution of the datasets corresponding to these cities and combine them with the social information provided in them. In particular, we will focus on the socio-economic status of individuals, a particularly important characteristic that appears in the specific context of the Colombian society, to compare the movements of individuals across different socio-economic classes.

## 2. Human mobility networks

Representing human mobility by networks imposes certain geometric conditions on nodes and edges, as it is spatially embedded in two- or three-dimensional spaces. This spatial nature has important effects on the topological properties of the network and on the processes that take place on it [13–15]. Besides, over a short period of time (from minutes to hours), there are many possible spatial scales for humans to travel, ranging from urban mobility within a few kilometres, to inter-urban or international trips within hundreds or thousands of kilometres. Thus, mobility studies often focus on a particular scale, be it global [16–18], continental [19], national [20] or regional [21,22]. Previous studies on Colombian mobility consider human flows across municipalities, or at a higher level of aggregation, say across metropolitan areas, [23], while in this paper, we are interested in an urban context, and specifically commuting networks.

Many commuting networks are based on mobility surveys or census data at different scales. De Montis *et al.* [24] analysed the inter-municipal scale commuting network of Sardinia by using mobility census data. They studied the relationship between the topological structure of networks with traffic and other urban-related indicators. Ramasco *et al.* [25] applied a weighted rich-club effect model in order to explore traffic organization in mobility networks. They applied the model to three mobility networks at different scales: United States (US) air transportation, and the daily commuting patterns in both US counties and Italian municipalities. Goetz *et al.* [26] examined the effects of the US commuting patterns on local economic growth. Caschilli & De Montis [20] analysed the commuting system of the USA at the county level, and they proposed measures of accessibility (based on travel cost and spatial interaction) according to complex network properties.

In addition to mobility surveys, human mobility networks can be constructed from different data sources. Chowell *et al.* [27] made large-scale simulations in a pseudo-agent-based model aimed at describing the displacement of individuals in the city of Portland, Oregon (USA). Brockmann *et al.* [28] used bank notes to study travel behaviour in the US and found that the distribution of trip distances decays as a power law. Lenormand *et al.* [29] used credit-card transaction records in Barcelona and Madrid as mobility proxies; they assessed the influence of socio-demographic characteristics on the way people move and spend their money. Further studies in human mobility used mobile phone calls as a proxy of mobility. González *et al.* [30] found that human trajectories show a high degree of temporal and spatial regularity by analysing the position of mobile phone users. Louail *et al.* [31] defined an urban dilatation index that measures the average distance between individuals and how it evolves during a day. Candia *et al.* [32] explored the interplay between human dynamics and human mobility by counting the number of callers that changed coordinates in a time window of 30 min. Recently, the literature has explored the potential of mobile phone records to estimate origin-destination matrices [33–36]; in particular, Coscia & Hausmann [37], based on Colombian cell-phone datasets, have recently shown that mobility networks can be faithfully obtained from cell-phone calls networks. More data sources used in the study of urban mobility are GPS traces [38], social media check-in data [39], taxi-traced datasets [40,41] and urban transportation smart and fare cards transactions [42].

## 3. Datasets

Our analysis of urban mobility is based on data collected from origin-destination surveys (ODS) in the Colombian cities of Manizales and Medellín [43–45]. The corresponding datasets are available in [46]. As discussed above, this kind of survey is one of the main instruments for gathering information about urban mobility along with other information about trips, travellers and households. ODS are home-based, i.e. each home member (older than 5 years old) is asked about the trips performed the workday before the interview or, as in the case of Manizales, they were asked about the trips they usually make during working days in a regular week. Collected data include the origin and destination zones, the departure and arrival times, the transportation mode used and the purpose of each trip. In addition, householders are characterized by their socio-economic characteristics, such as age, gender, occupation and characteristics of their housing, among others.

In table 1, we present and compare the characteristics of the ODS, such as the year of the survey, the number of origin-destination zones defined for each city, the area of the zone of study in each ODS, the population of the city or metropolitan area, that was used in the survey to calculate the sample size (rounded to the nearest hundred thousand), the total amount of trips reported by the interviewed and the total amount of trips of the city, taking into account the expansion factors assigned to each trip (rounded to the nearest ten thousand).

**Table 1.** General description of the origin-destination surveys of the cities of Medellín and Manizales.

	Medellín	Manizales
year of the survey	2006	2005
origin-destination zones	413	57
area (in kilometres)	1150	508
	(184 urban)	(45 urban)
population (millions of inhabitants)	3.5	0.4
sample size (people interviewed)	56 513	4089
trips reported in the sample	127 849	24 760
trips expanded to the population (in thousands of trips)	4000	550

Note that, as ODS are sample based, they must be expanded to represent the population or universe from which the sample was drawn [47]. Mobility studies based on ODS use the expansion factors in order to scale the results up to population [48–50]. In the ODS from Medellín and Manizales, every trip is associated with an expansion factor that depends of the transportation zone and the socio-economic strata; and the expanded sample was validated with population official information of planning and governmental agencies. In addition, the expansion factor for morning peak hour in Medellín was also validated by traffic counts on the main roads.

### 3.1. Socio-economic stratification

In Colombia, laws 142 and 143 of 1994 defined a system that classifies households according to their physical and environmental characteristics. This classification was to used to establish cross-class subsidies of public utilities services based on the criteria of solidarity, self-financing, redistribution and social and economic efficiency [51]. As a result, there were defined six socio-economic strata according to the public services utilities paying capacity; households in strata one to three are subsidized, stratum four householders pay the marginal cost of services; and those in strata five and six, along with the commercial and industrial sectors, pay more in order to contribute to the subsidiary system. Moreover, this stratification system is also used to set differential rates for taxes, public university tuition fees and other subsidies and services.

Although the overlap between these six official strata and the social class of the individuals is not perfect, this classification according to the household characteristics and the paying capacity has been widely used as a proxy of the socio-economic status of people in Colombia [51]. In this way, those people living in status 1 represent the lowest-income householders whereas those individuals in status 6 correspond to the wealthiest ones.

The distribution of households according to its socio-economic class and the number of sampled households for the ODS in Medellín is reported in table 2, as stated in the technical report of the survey [44]. In the case of Manizales’ ODS, the amount of sampled households is not explicitly described in the technical report of the survey, and what we show in table 2 for this issue is our own inference from the ID data in the survey. In both surveys, the samples were intended to represent proportionally the distribution of socio-economic strata in the population, which they approximately do, as can be seen in the percentages shown in table 2. Perhaps, a word of caution should be made here, namely that the ODS zones (that will correspond to nodes in our mobility networks defined below) do not necessarily coincide with the neighbourhoods associated with physical and environmental characteristics considered for socio-economic stratification. In fact, the ODS zones generally include households assigned to different strata.

**Table 2.** Distributions of sampled and officially recorded households according to their socio-economic stratum.

city		class 1	class 2	class 3	class 4	class 5	class 6
Medellín	sampled households	1422	10 125	9290	1549	916	359
	(percentage)	6%	42.8%	39.3%	6.5%	3.9%	1.5%
	official record	65 494	284 082	306 895	90 786	54 482	22 434
	(percentage)	7.9%	34.5%	37.2%	11%	6.6%	2.7%
Manizales	sampled households	174	773	1463	759	161	230
	(percentage)	4.9%	21.7%	41.1%	21.3%	4.5%	6.5%
	official record	5669	22309	44 069	12 882	2759	4319
	(percentage)	6.1%	24.2%	47.9%	14%	3%	4.7%

## 4. Results

We first analyse the structure and topology of daily urban mobility networks. To this aim, the city is divided into *N* areas (nodes) so that the mobility network is fully described by a *N*×*N* weighted origin-destination (OD) matrix **W**, with elements *W*_*ij*_ denoting the number of reported trips from area (node) *i* to area (node) *j*. Alternatively, one can use the adjacency matrix A, so that *A*_*ij*_=1 if at least one trip from *i* to *j* has been observed whereas *A*_*ij*_=0 otherwise. With these two matrices one can compute the typical characteristics of the nodes (areas) of the network such as the degree of a node *i*, i.e. the number of areas connected to *i*:

4.1 $$k_{i}=\sum_{j=1}^{N}A_{ij},$$

the strength of node *i*, i.e. the number of trips from node *i*:

4.2 $$s_{i}=\sum_{j=1}^{N}W_{ij},$$

the clustering coefficient of a node, i.e. the fraction of neighbours of *i* that are connected each other:

4.3 $$c_{i}=\frac{2}{k_{i}(k_{i}-1)}\sum_{j,l=1}^{N}A_{ij}A_{jl}A_{li},$$

or the shortest path from node *i* to node *j*, defined as the minimum sequence of *n* nodes, (*i*,*l*_1_,*l*_2_,…,*l*_*n*−1_,*j*), so that *A*_*i*,*l*_1__=*A*_*l*_1_*l*_2__=⋯=*A*_*l*_*n*−1_*j*_=1, which also defines the distance between *i* and *j* as *d*_*ij*_=*n*.

**Figure 1.**
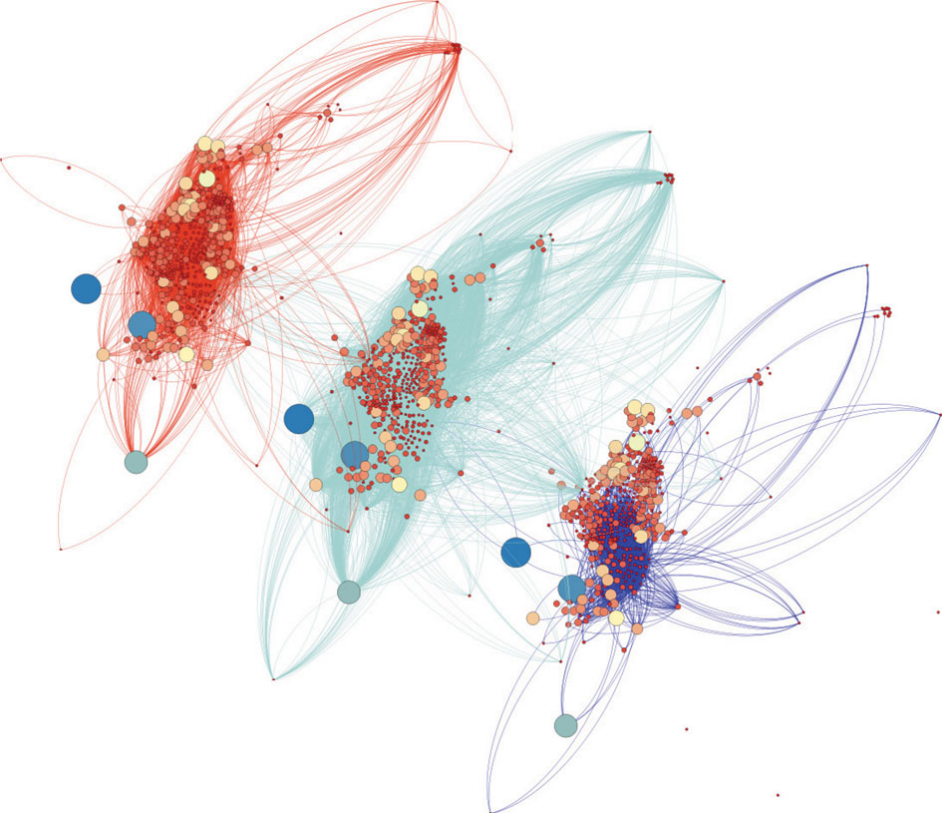
Three mobility networks of the city of Medellín corresponding to three different socio-economic strata. From left to right status 1 (poorest), status 3 (middle class) and status 6 (richest). Nodes represent the origin and destination zones according to the ODS, sizes of nodes show their strength and colours represent their degree.

As anticipated above, we are interested in analysing the mobility networks of the six different socio-economic classes present in the cities of Medellín and Manizales. In figure 1, we show, for the city of Medellín, a representation of the mobility networks corresponding to classes 1, 3 and 6 (poor, middle-class and rich individuals, respectively). Note that, in principle, these networks should contain the same set of *N* nodes, because nodes account for the areas of the city. However, it might be the case that individuals from class *α* (*α*=1,…,6) neither live nor visit a particular set of areas of the city. In this case, the effective number of non-isolated nodes of the mobility network of class *α* is *N*^*α*^<*N*.

Each of the six mobility networks are encoded in different adjacency and weighted OD matrices, **A**^*α*^ and **W**^*α*^, and thus they display different structural properties. In tables 3 and 4, we present the main properties of the mobility networks of Medellín and Manizales, respectively. We show both, the properties of the network comprising all the movements in the city (regardless of the socio-economic class), and those of the subnetworks corresponding to the each socio-economic class *α*. In particular, we show the total number of (non-isolated) nodes and edges and the averages (over the set of non-isolated nodes) of the measures introduced above. Namely, the average degree of the mobility network of class *α* is

4.4 $$\langle k^{\alpha }\rangle =\frac{1}{N^{\alpha }}\sum_{i=1}^{N^{\alpha }}k_{i}^{\alpha },$$

the average strength:

4.5 $$\langle s^{\alpha }\rangle =\frac{1}{N^{\alpha }}\sum_{i=1}^{N^{\alpha }}s_{i}^{\alpha },$$

the average clustering coefficient:

4.6 $$\langle c^{\alpha }\rangle =\frac{1}{N^{\alpha }}\sum_{i=1}^{N^{\alpha }}c_{i}^{\alpha },$$

and, finally, the average path length:

4.7 $$\langle d^{\alpha }\rangle =\frac{2}{N^{\alpha }(N^{\alpha }-1)}\sum_{i=1}^{N^{\alpha }}\sum_{j>i}d_{ij}^{\alpha }.$$;

**Table 3.** Network properties of a work-day urban mobility in the city of Medellín together with those of the subnetworks corresponding to the mobility of the six socio-economic classes.

	total	class 1	class 2	class 3	class 4	class 5	class 6
nodes (*N*^*α*^)	413	337	408	391	302	258	195
edges	34 225	3042	15 085	16 197	4564	2925	1297
〈*k*^*α*^〉	78.87	9.03	36.97	41.43	15.11	11.34	6.65
〈*s*^*α*^〉	11 567.61	883.03	4035.50	4462.89	1751.57	1352.55	1079.16
〈*c*^*α*^〉	0.439	0.176	0.303	0.361	0.286	0.338	0.312
〈*d*^*α*^〉	1.832	2.833	2.119	2.144	2.518	2.558	2.693

**Table 4.** Network properties of a work-day urban mobility in the city of Manizales together with those of the subnetworks corresponding to the mobility of the six socio-economic classes.

	total	class 1	class 2	class 3	class 4	class 5	class 6
nodes (*N*^*α*^)	57	51	56	56	54	46	45
edges	2571	359	1171	1570	936	290	390
〈*k*^*α*^〉	45.11	7.04	20.91	28.04	17.33	6.3	8.67
〈*s*^*α*^〉	9254.12	526.88	2090.71	4475.29	1515.91	444.5	680.07
〈*c*^*α*^〉	0.850	0.331	0.495	0.717	0.678	0.547	0.641
〈*d*^*α*^〉	1.211	2.256	1.658	1.510	1.732	2.180	1.932

From both tables, it is clear the differences between the mobility networks of the different socio-economic classes. In particular, the most populated classes (2 and 3) display the most densely connected networks as shown from the values of their average degree 〈*k*〉 and average strength 〈*s*〉 and their relatively small average path lengths 〈*d*〉 compared with those of the other subnetworks. Importantly, the less populated classes 1 and 6 display important differences rooted in their socio-economic distance such as the larger average path length of class 1 and the remarkably larger average strength and clustering of class 6. These differences reveal that trips in class 6 are highly redundant and localized whereas for class 1 the displacement is highly dispersed across the city. These different networks were structurally characterized in detail in [52], where the changes and variations across socio-economic strata and transportation modes were analysed under the perspective of multilayer complex networks [53]. The results in this multilayer analysis show different behaviours according to the socio-economic strata; low-income householders (socio-economic strata 1 and 2) show segregation in their mobility patterns; middle-income travellers (strata 3 and 4) show a multimodal mobility, and high-income people (strata 5 and 6) perform their trips to a few zones of the urban area using costly modes.

### 4.1. Temporal patterns of urban mobility

**Figure 2.**
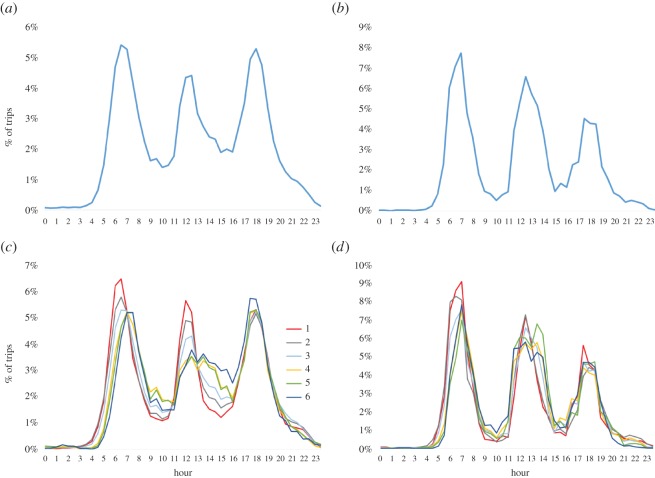
Percentage of trips as a function of time during a week day. Time is discretized in intervals of 30 min. Left panels (*a*) and (*c*) correspond to the city of Medellín, while right panels (*b*) and (*d*) correspond to Manizales. Lower panels show separately this percentage for each socio-economic class, and upper panels do that for the whole population.

We now focus on the temporal dependence of the trips performed in the cities of Medellín and Manizales. In figure 2*a*,*b*, we show the number of trips observed in the two cities as function of time during a week day. In particular, we have computed, for a time window of 30 min, the percentage of trips performed in this window with respect to the total trips observed during the day. It becomes clear that there are three peak windows: early morning (6–8), midday (12–14) and evening (18–20). These three peaks, together with two periods of intermediate activity (8–12 and 14–18) and a large period of low activity (20–6), conform the urban circadian rhythms of the two cities.

The information provided in the mobility surveys allow us to disentangle the daily rhythm of each socio-economic class from the rest of the population. In panels (*c*) and (*d*) of the same figure 2, we show the temporal patterns of the movements of the six socio-economic classes by computing the percentage of trips of class *α* in each time window of 30 min with respect to the total trips performed by the individuals of this class during the day.

From these panels, one observes that the temporal patterns vary across the socio-economic classes and, surprisingly, in most of the cases, this variation follows one-by-one the wealth ordering of these classes. First, for both cities, we observe that the early-morning peak delays as the class increases, i.e. the onset for the this peak appears first for classes 1 and 2 around 5.00–6.00, while this onset is delayed until 7.00 for class 6. In addition, especially for the city of Medellín, for the second peak appearing at midday we observe that the shape and height of the peak depends crucially on the wealth of the class. Namely, for class 1 the peak is highly localized around 12.00 whereas, as the wealth increases, it appears smaller and broader. This becomes clear between 13.00 and 16.00 where the activity increases with the wealth of the class, from 1 to 6. For the city of Manizales, we also observe differences in the second peak, manifested by a broadening of the activity around 12.00 as the class increases. Finally for the third peak, we do not observe significant differences among socio-economic classes.

### 4.2. Spatio-temporal patterns of urban mobility

We now analyse the gross characteristics of the spatial localization of both origins and destinations of the reported urban movements. Here, we restrict our attention to the city of Medellín, a much more populated city than Manizales, this last deserving a somewhat specific analysis owing to its small size. In this preliminary approach, we perform an aggregation of the temporal data in three time slots, namely AM (from 4.00–10.00), MD (from 10.00–16.00) and PM (from 16.00–22.00). Almost no information is lost by the exclusion of the overnight interval (22.00–04.00) because, for all the socio-economic classes, this time interval shows a negligible trip activities. However, although the choice of three time slots (instead of, e.g. four or other) is a reasonable compromise, favoured by the temporal pattern shown in figure 2, there is clearly some loss of information due to the time discretization performed.

**Figure 3.**
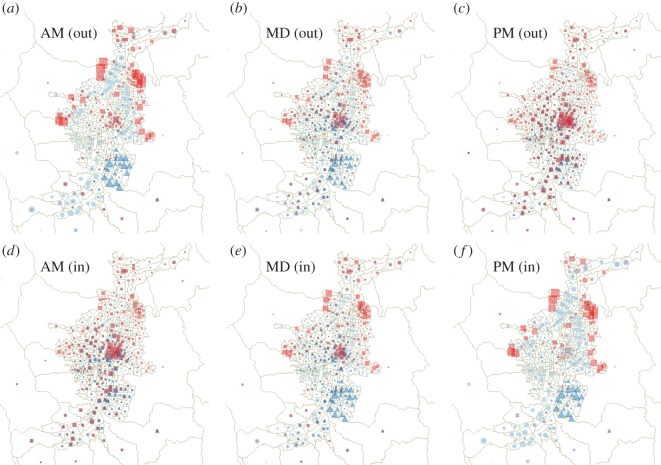
Geographical location of origin (out, (*a*–*c*)) and destination (in, (*d*–*f*)) nodes of the trips performed during each time slot (AM left, MD middle, PM right) by the individuals of three socio-economic classes, namely 1 (poorest, red squares), 3 (low middle class, light blue circles) and 6 (richest, dark blue triangles). The size of symbols indicate the in- (out-)strength of nodes for the time slot considered.

In figure 3, we show the geographical location of origin (out) and destination (in) nodes of the trips performed during each time slot by the individuals of three socio-economic classes, namely 1 (poorest), 3 (low middle class) and 6 (richest). The patterns corresponding to the rest of the classes roughly interpolate between those of these three chosen classes. The size of the symbols in the figure represents the percentage of trips within the corresponding class having its origin or destination in the corresponding node, i.e. the (in- or out-) strength of the node, for the time slot. Note that the average strength shown in table 2 corresponds to the whole day, and for this time interval the in-strength and out-strength of nodes coincide.

Panels AM (out), at the top left, and PM (in), at the bottom right, are almost exactly the same because of the negligible overnight trip activities, so that they provide a sort of snapshot of the geographical distribution at night of the three classes considered. Also, the strong resemblance of panels AM (in), at the bottom left, and PM (out), at the top right, would indicate a very similar geographical distribution of permanence for each class during morning and afternoon periods, except for class 6, for which the broadness of the intermediate peak of mobility seen in panel (*c*) of figure 2, blurs somehow the notion of afternoon period of staying.

Class 3 shows more geographical dispersion of strength of origin-destination nodes around the city than the other classes shown in figure 3. This could have been guessed from (as it is partially captured by) the large number of edges (see second row in table 2) corresponding to the class 3 network. This observation on the strength dispersion of class 3 is naturally related to the fact that this class is more populated than classes 1 and 6; however, the latter fact might not necessarily exhaust the explanation of the observation. By contrast, the strength of origin-destination nodes for the upper class 6 is remarkably localized. Moreover, more than half of the city nodes seem to be unvisited by individuals of the upper class in this dataset, a fact that cannot be solely explained by the smaller size of this subpopulation. Regarding class 1, strength changes from being very concentrated at peripheral areas overnight (see panel AM (out) or PM (in)) to disperse over almost everywhere but with a compact big spot in the geographical centre of the city at morning and afternoon staying periods.

### 4.3. Efficiency of urban mobility

The differences in the temporal patterns of mobility across socio-economic classes may be rooted in different features such as the geographical location of individuals, the transportation means or the purposes of the trips. To shed light on this issue, we now analyse the efficiency of the movements performed by the individuals of each of the social compartments during a day.

**Figure 4.**
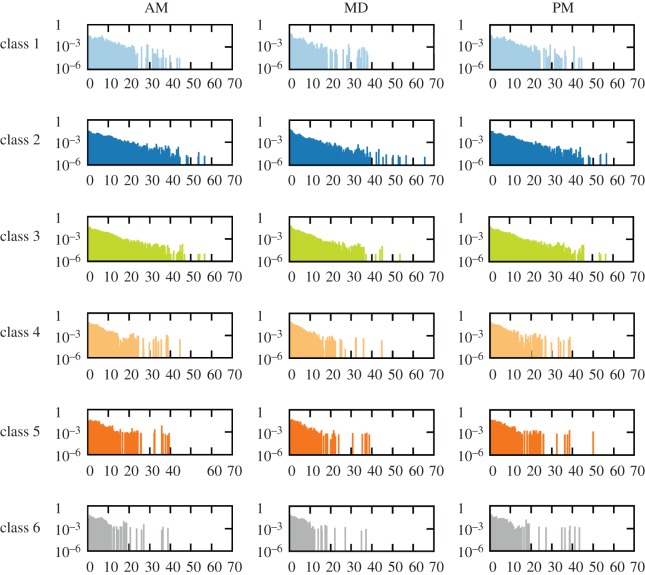
Distribution of the length *d* (in kilometres) of the trips, *P*(*d*), performed by each socio-economic class for each of the time intervals AM, MD and PM.

In figure 4, we report, for the city of Medellín, the distributions of the trip distances (in kilometres), *P*(*d*), of each socio-economic class for the three time intervals AM, MD and PM. From the panels, we observe that the distribution of displacements *P*(*d*) decay with *d* for small distances (*d*<10 km). For long distances (*d*>10 km) persists for socio-economic classes 2 and 3. However, for classes 4, 5 and 6, the initial decay is followed by a significantly large number of long distance trips, making the distribution *P*(*d*) moderately flat for *d*>10 km.

To better quantify the length of the displacements of each socio-economic class in table 5, we show the average distance 〈*d*〉 travelled by each socio-economic class for the three time intervals (AM, MD and PM). From the table, we clearly observe that, for classes 4, 5 and 6, the average length of trips increases with the socio-economic status regardless of the time interval. This result clearly correlates with the significant number of trips in the tails of the of the distributions *P*(*d*). In contrast for classes 1, 2 and 3, the situation is not that simple and depends on the particular time interval. Interestingly, for AM and PM, class 2 displays the largest value of 〈*d*〉 among all the classes.

**Table 5.** Average values for the distances of trips (in kilometres), travel times (in minutes), and the effective velocity (in kilometres per hour) for each time interval (AM, MD and PM) and socio-economic class in the city of Medellín.

		class 1	class 2	class 3	class 4	class 5	class 6
〈*d*〉 (km)	AM	4.82	5.14	4.70	3.98	4.66	4.95
	MD	2.83	3.19	3.36	3.18	3.41	3.87
	PM	4.78	4.98	4.58	3.93	4.35	4.84
〈*τ*〉 (min)	AM	35.22	32.56	29.26	25.70	24.71	25.92
	MD	28.14	26.30	25.49	23.68	22.52	24.16
	PM	39.97	35.33	31.96	27.07	26.19	27.73
*v*_eff_ (km h^−1^)	AM	8.211	9.471	9.638	9.292	11.315	11.458
	MD	6.034	7.278	7.909	8.057	9.085	9.611
	PM	7.175	8.457	8.598	8.711	9.965	10.472

Another interesting feature shown in table 5 is the average time spent per trip 〈*τ*〉. In general, 〈*τ*〉 decreases as the socio-economic class increases from 1 to 5. However, class 6 always shows values of *τ* in between those of classes 3 and 4. Finally, we can use the information provided by 〈*d*〉 and 〈*τ*〉 to derive an effective velocity *v*_eff_=〈*d*〉/〈*τ*〉 that quantifies the efficiency of the mobility across socio-economic classes. The values of *v*_eff_ are shown in the last three rows of table 5. In this case, we obtain a more clear view about the importance of socio-economic classes in the mobility because, regardless of the time interval, the value of *v*_eff_ increases with the wealth of the socio-economic class. This points to the efficiency of the mobility as a key factor behind the differences observed in the temporal activity patterns.

The former results are in agreement with the case of Manizales (table 6) where, despite small deviations for class 6, one can conclude that 〈*d*〉 increases with socio-economic wealth, whereas 〈*τ*〉 decreases. More importantly, as in the case of Medellín, mobility efficiency (quantified by *v*_eff_) increases with wealth.

**Table 6.** Average values for the distances of trips (in kilometres), travel times (in minutes), and the effective velocity (in kilometres per hour) for each time interval (AM, MD and PM) and socio-economic class in the city of Manizales.

		class 1	class 2	class 3	class 4	class 5	class 6
〈*d*〉 (km)	AM	2.147	2.196	2.378	2.44	2.610	2.574
	MD	1.497	1.805	1.910	2.298	2.535	2.500
	PM	2.352	2.633	2.479	2.515	2.856	2.660
〈*τ*〉 (min)	AM	28.54	28.99	25.77	24.19	23.68	26.04
	MD	24.40	26.50	24.07	24.51	22.63	22.67
	PM	31.34	33.36	27.50	26.52	25.71	25.19
*v*_eff_ (km h^−1^)	AM	4.513	4.545	5.536	6.052	6.613	5.931
	MD	3.716	4.087	4.7611	5.625	6.721	6.617
	PM	4.503	4.736	5.409	5.690	6.665	6.336

## 5. Conclusion

We have presented a dataset about the human mobility in the urban areas of Medellín and Manizales incorporating three important ingredients: (i) the geographical distribution of origin and destination of the trips, (ii) the temporal pattern of these trips, and (ii) the information about the socio-economic status of the individuals. Although the role of the two first ingredients have been extensively studied in the literature about urban mobility, the information about socio-economic class provides us with a novel dimension to be correlated with spatial and temporal ones. Exploiting these three ingredients, the aim of this article has been to describe the spatio-temporal patterns shown by the different socio-economic compartments.

The first conclusion of the work is that the mobility networks associated with each of the socio-economic classes are quite different. For instance, that of the poorest compartment (class 1) resembles a tree-like graph, by covering most of the city area with a relatively poor wiring and a small level of redundancy (pointed out by the small clustering coefficient). By contrast, compartments 2 and 3 cover efficiently most of the areas of the city displaying dense mobility networks. As the wealth of the individuals increases (classes 4, 5 and 6), the number of covered areas decreases and the spatial patterns of movements becomes more localized, this yielding an increase of the clustering coefficient. These results are confirmed by the spatial distribution of in and out areas for class 6, when confronted with those of classes 1 and 3.

Another important feature that varies across socio-economic classes is the temporal distribution of the trips. The three peaks observed for the total number of trips unveil different patterns associated with the wealth of the individuals. Namely, we have shown that the onset and localization of the early morning peak delays as the wealth of the socio-economic class increases.

This result should be related with the transportation efficiency of rich classes that, although displaying long distance trips, show a larger effective velocity than poor classes. In addition, the midday activity also reveals that poor classes highly localize their movements around 00.00 whereas the activity of richer compartments spreads across a broad range of afternoon hours.

As a conclusion, we have shown the importance of incorporating the differences in the spatio-temporal organization of the mobility patterns according to the socio-economic status in the studies about human mobility and its related processes. As an example, it would be of interest to incorporate the presence of socio-economic differences when studying the development of contagion processes or the dissemination of information in urban areas. On the other hand, we note that the particular structure of the Colombian society, being compartmentalized in six socio-economic strata and showing a highly unequal wealth distribution, highlighting the heterogeneity of urban mobility patterns. Thus, it is necessary to complement this study with others in different social settings so to have a general picture about the influence of wealth status in urban mobility patterns.

## Data accessibility

Datasets used in this study are available via the Dryad Digital Repository (doi:10.5061/dryad.hj1t4)[46].
